# Machine learning-based prediction model for emergency department visits using prescription information in community-dwelling non-cancer older adults

**DOI:** 10.1038/s41598-023-46094-z

**Published:** 2023-11-02

**Authors:** Soyoung Park, Changwoo Lee, Seung-Bo Lee, Ju-yeun Lee

**Affiliations:** 1 College of Pharmacy and Research Institute of Pharmaceutical Sciences, Seoul National University, Seoul, 08826 Republic of Korea; 2 Department of Transdisciplinary Medicine, Seoul National University Hospital, Seoul, 03080 Republic of Korea; 3 Department of Medical Informatics, Keimyung University School of Medicine, Daegu, 42601 Republic of Korea; 4 Department of Medical Device Development, Seoul National University College of Medicine, Seoul, 03080 Republic of Korea

**Keywords:** Geriatrics, Population screening

## Abstract

Older adults are more likely to require emergency department (ED) visits than others, which might be attributed to their medication use. Being able to predict the likelihood of an ED visit using prescription information and readily available data would be useful for primary care. This study aimed to predict the likelihood of ED visits using extensive medication variables generated according to explicit clinical criteria for elderly people and high-risk medication categories by applying machine learning (ML) methods. Patients aged ≥ 65 years were included, and ED visits were predicted with 146 variables, including demographic and comprehensive medication-related factors, using nationwide claims data. Among the eight ML models, the final model was developed using LightGBM, which showed the best performance. The final model incorporated 93 predictors, including six sociodemographic, 28 comorbidity, and 59 medication-related variables. The final model had an area under the receiver operating characteristic curve of 0.689 in the validation cohort. Approximately half of the top 20 strong predictors were medication-related variables. Here, an ED visit risk prediction model for older people was developed and validated using administrative data that can be easily applied in clinical settings to screen patients who are likely to visit an ED.

## Introduction

The rate of emergency department (ED) visits has been increasing faster than the population growth rate^1^. According to the results of previous studies conducted in the United States (U.S.), 144.8 million people in the U.S. visited the ED in 2017 and the total expenditure was $76.3 billion^2^. The rate of ED visits by elderly people aged 75 years or older in 2018 was 60 visits per 100 persons, which was higher than that for all other groups (overall, 40 visits per 100 persons), except for infants under the age of 1 year (101 visits per 100 infants)^3^. Multiple comorbidities and polypharmacy in older adults increase the risk of medication-related ED visits. Jeon et al.^4^ identified the risk of hospitalization or ED visits in older patients and found that those taking one or more general potentially inappropriate medications (PIMs) based on the Beers criteria had a twofold increased risk of ED visits than those not taking PIMs. A study of patients who visited the ED in the U.S. from 2017 to 2019 found that approximately 6.1 ED visits per 1000 people per year were due to medications^5^. In addition, 38.6% of drug-related ED visits required follow-up hospitalization, and the hospitalization rate of those aged ≥ 75 years was 48.9%. As such, ED visits related to medication use in elderly people are significantly higher than those in other age groups; therefore, countermeasures are needed.

One possible approach to reduce the number of ED visits by older patients is to identify patients likely to require an ED visit and proactively adjust for modifiable factors, such as medication use. Several studies have been conducted to predict the risk of requiring an ED visit. One such study conducted in the U.S., using an electronic administrative database, predicted ED visits of community-dwelling older adults using demographic and underlying disease data, but did not include modifiable variables^6^. Hippisley and Coupland^7^ also predicted emergency hospitalization with primary care data for patients aged 18–100 years using demographic and lifestyle, comorbidity, clinical, and medication data. However, the only modifiable variable in the final model, the medication variable, was limited to six medication classes. In addition, in a study conducted in Scotland, the Predicting Emergency Admissions Over the Next Year (PEONY) score was used to predict the emergency admissions of patients aged ≥ 40 years using chronically used drugs. However, predictions were based on prescriptions from the past 3 years instead of only including medication used recently, making it difficult to use^8^. Therefore, unlike other variables, it is necessary to predict ED visits using readily available and modifiable medication use variables, such as replacement with other drugs or discontinuing their use when unnecessary.

Logistic regression has traditionally been used to develop a predictive model for ED visits in community-dwelling patients. The prediction model using National Health Service claims data from the United Kingdom included 89 variables with multivariable linear regression^9^. In addition, a study of community-dwelling patients aged ≥ 60 years predicted hospitalization and ED visits by calculating risk scores using regression estimates^6^. According to previous studies, applying machine learning (ML) methods to predict similar outcomes, such as readmission, showed improved results^10,11^. However, ML approaches have not been widely used to predict ED visits, as few studies have analyzed the relationship between medication use and ED visits. Therefore, the present study aimed to develop and validate a model that could predict the risk of ED visits among older adults using extensive medication variables, including those generated by explicit clinical prescribing criteria for elderly people from nationwide claims data by applying various ML methods.

## Methods

### Study population

**Figure 1:**
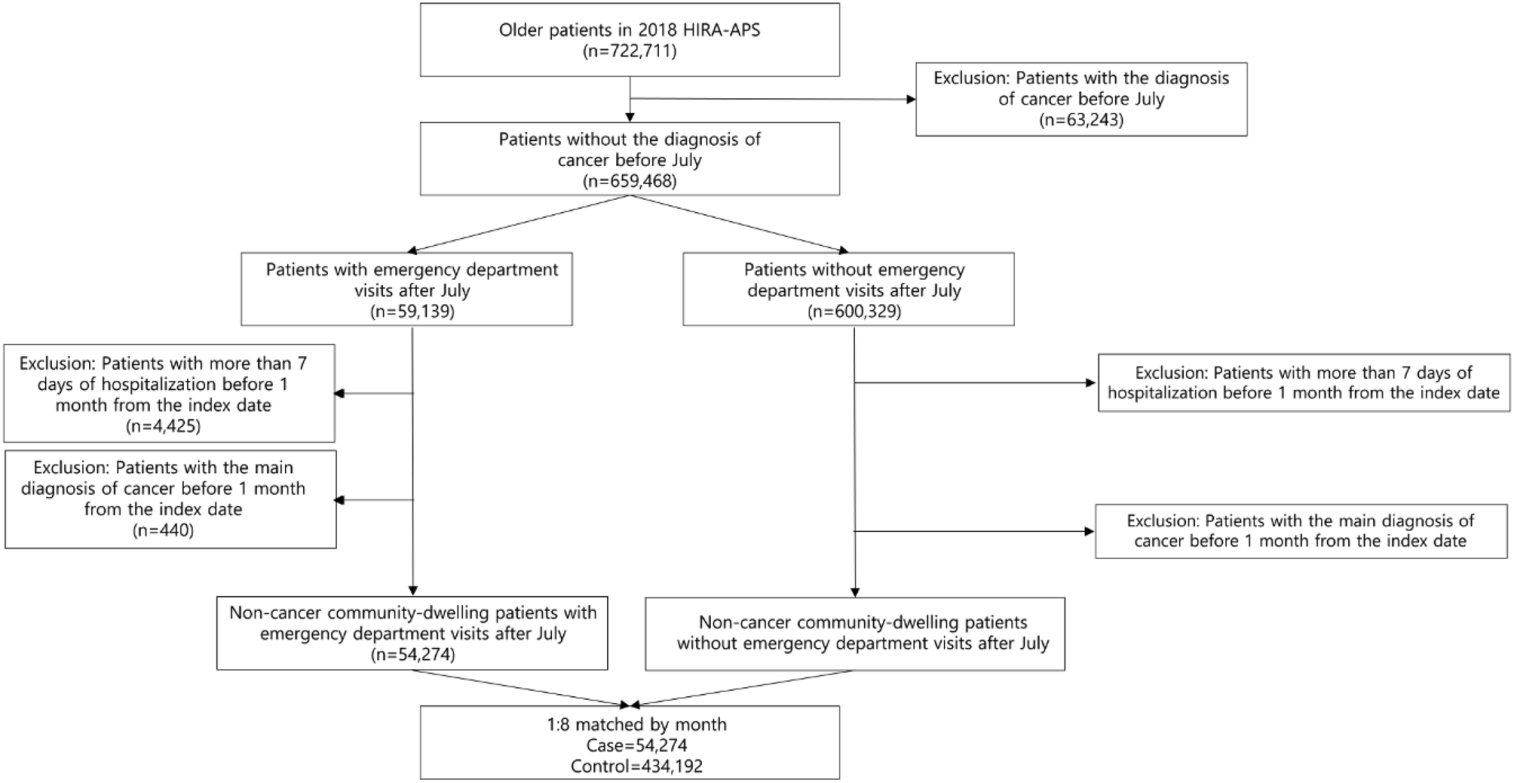
Patient selection flow for the training set.

Two years of nationwide claims data from the Health Insurance Review and Assessment Service-National Adult Patients Sample (HIRA-APS) were used, which included those aged 65 years or older, with 2018 as a training set and 2019 as a test set. In Korea, the national health insurance system covers a vast majority, approximately 98% of the population, and the Health Insurance Review and Assessment (HIRA) database contains claims data for over 90% of the population, ensuring the generalizability of our analysis. The HIRA-APS dataset, which we utilized, is a 10% stratified random sample of claims data for individuals aged over 65 years, providing comprehensive information on patient demographics, disease diagnoses based on the International Statistical Classification of Diseases Tenth Revision (ICD-10), procedures, and prescription details^12^. Patients with a main diagnosis of cancer before July were excluded because it was thought that there would be differences in the reasons for visiting the ED between cancer patients and noncancer patients. For the case group, the first ED visit after July, without a main diagnosis of cancer, was included, and the ED visit date was defined as the index date. Controls were selected from among those who did not visit the ED after July. To reduce the monthly difference between selected patients, matching was performed monthly, and the first outpatient visit date of the matched month was defined as the index date for the controls. To account for the increasing complexity and poorer health status observed in recent hospitalized adults, we excluded patients who had been hospitalized for more than 7 days in the month prior to the index date (Fig. 1). This study was approved by the Seoul National University Institutional Review Board (IRB No. E2112/001-001). The informed consent from the participants was waived by the Seoul National University Institutional Review Board because this study used de-identified data retrospectively. All methods were performed according to relevant guidelines and regulations.

### Outcome definition and measure

The data for analysis included three categories of variables, and the total number of variables was 146: (1) six sociodemographic characteristics of age, sex, insurance status, frailty score, history of ED visits, and hospitalization 3 months before the index date; (2) 32 comorbidities identified by the International Classification of Diseases, 10th edition (ICD-10) codes; and (3) 98 medication variables comprising 19 general PIMs, potentially inappropriate drug-drug interactions (DDI PIMs), disease-specific PIMs, and 34 potentially high-risk medication classes that were not PIMs. Medication use was assessed during the previous month based on the index date. PIMs were categorized as general PIMs, DDI PIMs, or disease-specific PIMs based on the Beers criteria and Screening Tool of Older Person’s Prescriptions (STOPP) criteria version 2^13,14^. Medication variables were collectively defined as overall medications (Supplementary Table 1). The anticholinergic burden was measured according to the Korean Anticholinergic Burden Scale (K-ABS), and frailty scores were measured according to the methods of a previous study^15,16^. Polypharmacy was defined as the maximum number of medications taken concomitantly over the past month.

The baseline model used in this study incorporated variables that were identified from previous studies conducted on community-dwelling patients^6,7^. However, due to the nature of the present study relying on claims data, certain variables such as smoking status, alcohol status, marital status, ethnicity, and laboratory test results could not be determined and thus were not included in the baseline model. Therefore, the baseline model included 10 variables: age, history of ED visits, hospitalization 3 months before the index date, diabetes, myocardial infarction, heart failure, ischemic heart disease, stroke, chronic obstructive pulmonary disease, and mental disorder.

### ML-based ED visit prediction

Eight ML prediction models were constructed: (1) logistic regression with ridge regularization^17^, (2) linear discriminant analysis (LDA)^18^, (3) random forest^19^, (4) XGBoost^20^, (5) LightGBM^21^, (6) CatBoost^22^, (7) deep neural network (DNN)^23^, and (8) TabNet^24^. Ridge regularization is one of the models that shrinks regression coefficients close to zero, thereby effectively selecting important predictors and improving the interpretability of the model. LDA is a method of performing classification by maximizing the variance between classes and minimizing the variance within classes. A random forest is an ensemble of decision trees from bootstrapped training samples, and random samples of a certain number of predictors are selected for tree induction. XGBoost, LightGBM, and CatBoost are gradient-boosted decision tree models that are also ensemble methods that construct new tree models predicting the errors and residuals of previous models^25^. When adding new models, this model uses a gradient descent algorithm to minimize the loss function. DNN is a type of artificial neural network, which is a model composed of multiple layers. It consists of an input layer, a hidden layer, and an output layer, and is used to solve nonlinear problems^26^. Each hidden layer adds nonlinearity through an activation function and is learned through a backpropagation algorithm^27^. TabNet is a deep learning model designed for efficient feature selection and prediction in tabular data. It utilizes a decision tree-like architecture, combines feature selection and prediction, and incorporates reinforcement learning for automatic feature selection, making it highly effective for various tabular data tasks. In all experiments, hyperparameter tuning of all ML analyses was performed via grid search.

**Figure 2:**
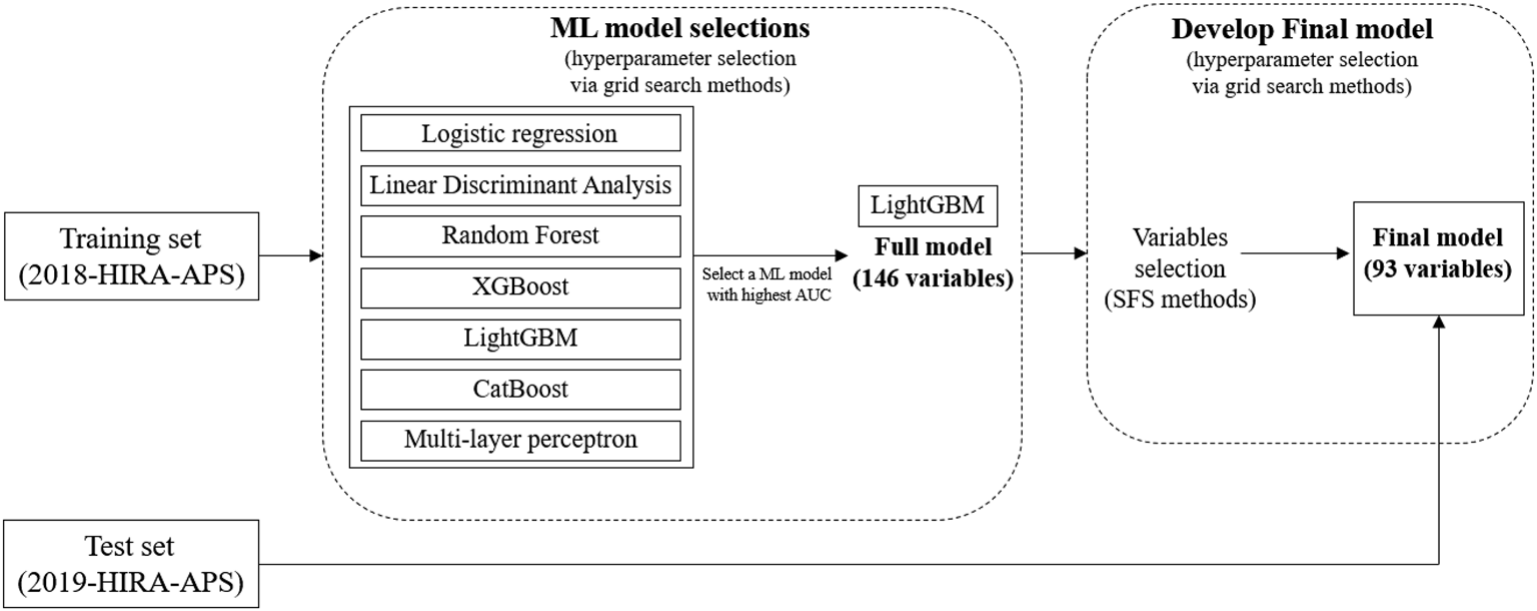
A flow chart for the process of the final model.

The ML models were trained using a training set with fivefold cross-validation. The performance was then evaluated using an independent test set after validation data in fivefold. To compare the trained models, the area under the receiver operating characteristic (ROC) curve (AUROC), accuracy, sensitivity, specificity, positive predictive value (PPV), negative predictive value (NPV), and F1-score were calculated. The development of the final model can be divided into two stages. In the first stage, hyperparameters were optimized using grid search for each machine learning (ML) model. Among the various ML models, we selected the one that demonstrated the best performance based on the AUC metric. In the second stage, the selected ML model was trained using variables that were chosen through sequential feature selection (SFS) methods (Fig. 2). In addition, the performance of the model was evaluated in the test set using AUROC, sensitivity, specificity, PPV, NPV, and F1-score. The hyperparameter tuning of all ML analyses was performed via grid search and the hyperparameter in which the range of values for each hyperparameter and the optimal values can be found in Supplementary Tables 3 and 4. All ML analyses were performed using Python version 3.8.8 and scikit-learn version 0.24.1^28^.

### Model interpretation

For model interpretation, Tree Explainer was utilized based on Shapley Additive Explanations (SHAP) values^29^. SHAP values were visualized in two ways: one was a plot of the mean of the absolute SHAP values, which indicated the average impact of each feature on the model output. The other was a scatterplot of the distribution of SHAP values, which indicated the tendency of each feature on the model output. The colors of each feature spectrum (according to the color bar: blue for low feature values and red for high feature values) indicated the feature value, that is, the intensity value of the respective feature in the spectrum.

### Statistical analysis

Cutoffs for ML models could be selected based on the preferred trade-off between sensitivity and specificity. For this study, potential candidate cutoffs and corresponding sensitivity and specificity were calculated for the model with the highest AUROC based on the Youden J statistic^30^. Based on these candidate cutoffs, false positives and negatives were inspected for gross patterns in the major variables. The Youden index was used to determine the cutoff threshold for each model. Delong’s test was used to compare the ROC curve between models^31^. Additionally, we assessed performance across various subgroups, including sex, age, frailty score, insurance status, ED visits, hospitalization, and comorbidity, using the area under the receiver operating characteristic curve. A descriptive analysis of the baseline characteristics of the study population was conducted using SAS version 9.4 (2017 SAS Institute, Cary, North Carolina, USA).

## Results

### Population characteristics

Among older adults in the 2018 and 2019 HIRA-APS, 54,274 and 56,762 community-dwelling patients visited the ED after July and were enrolled as cases, respectively. After monthly 1:8 matching, 488,466 and 510,858 patients were enrolled in the training set (2018-HIRA-APS) and test set (2019-HIRA-APS), respectively. The proportions of male patients were 40.8% and 41.0% in the training and test sets, respectively. In both the training and test sets, 1.6% of patients had visited the ED in the previous 3 months, and 4.2% had been hospitalized during the same period. Although there are statistically different in some comorbidities between the training and test set due to the large sample size, the proportion of patients diagnosed with hypertension, mental disorders and diabetes were roughly similar, with approximately 66%, 38% and 34%, respectively (Table 1, Supplementary Table 5).

**Table 1:** Patient’s characteristics of the training set and test set.

Variables	Training set	Test set	*p* value
Sociodemographic			
Age			< 0.001
65–69	158,038 (32.4)	162,982 (31.9)	
70–74	122,435 (25.1)	128,708 (25.2)	
≥ 75	207,993 (42.6)	219,168 (42.9)	
Insurance status			< 0.001
Health insurance	446,973 (91.5)	468,231 (91.7)	
Medical aid	41,493 (8.58)	42,627 (8.3)	
Sex			
Male	199,550 (40.9)	209,498 (41.0)	0.11
Frailty score			< 0.001
< 5	413,936 (84.7)	455,335 (89.1)	
5 to ≤ 10	54,878 (11.2)	49,895 (9.8)	
> 10	19,552 (4.0)	5627 (1.1)	
ER visits (previous 3 months)	7902 (1.6)	8362 (1.6)	0.45
Hospitalization (previous 3 months)	20,539 (4.2)	21,468 (4.2)	0.27
Comorbidity			
Hypertension	321,762 (65.9)	337,406 (66.1)	0.06
Mental disorder	186,933 (38.3)	198,268 (38.8)	< 0.01
Diabetes	166,261 (34.0)	179,417 (35.1)	< 0.01
COPD	114,078 (23.4)	114,841 (22.5)	< 0.01
Peptic ulcer disease	108,059 (22.1)	108,997 (21.3)	< 0.01
Liver disease	93,250 (19.1)	104,711 (20.5)	< 0.01
Ischemic heart disease	72,578 (14.9)	76,215 (14.9)	0.39
Asthma	70,720 (14.5)	70,044 (13.7)	< 0.001
Stroke	44,779 (9.2)	47,178 (9.2)	0.24
Anemia	34,298 (7.0)	38,153 (7.47)	< 0.001

*ED* emergency department, *COPD* chronic obstructive pulmonary disease.

### Development of the ED visit prediction model

Among the eight ML algorithms with all 146 variables, the random forest model had the lowest AUROC (0.658), whereas LightGBM (0.687) had the highest AUROC in the training set. To optimize the model, the LightGBM model (full model) was adopted. Supplementary Table 2 shows the performance of each ML model using all variables considered in this study. The range of values for each hyperparameter and the optimal values were presented in Supplementary Table 3 and 4.

### ED visit prediction performance in the final model

**Figure 3:**
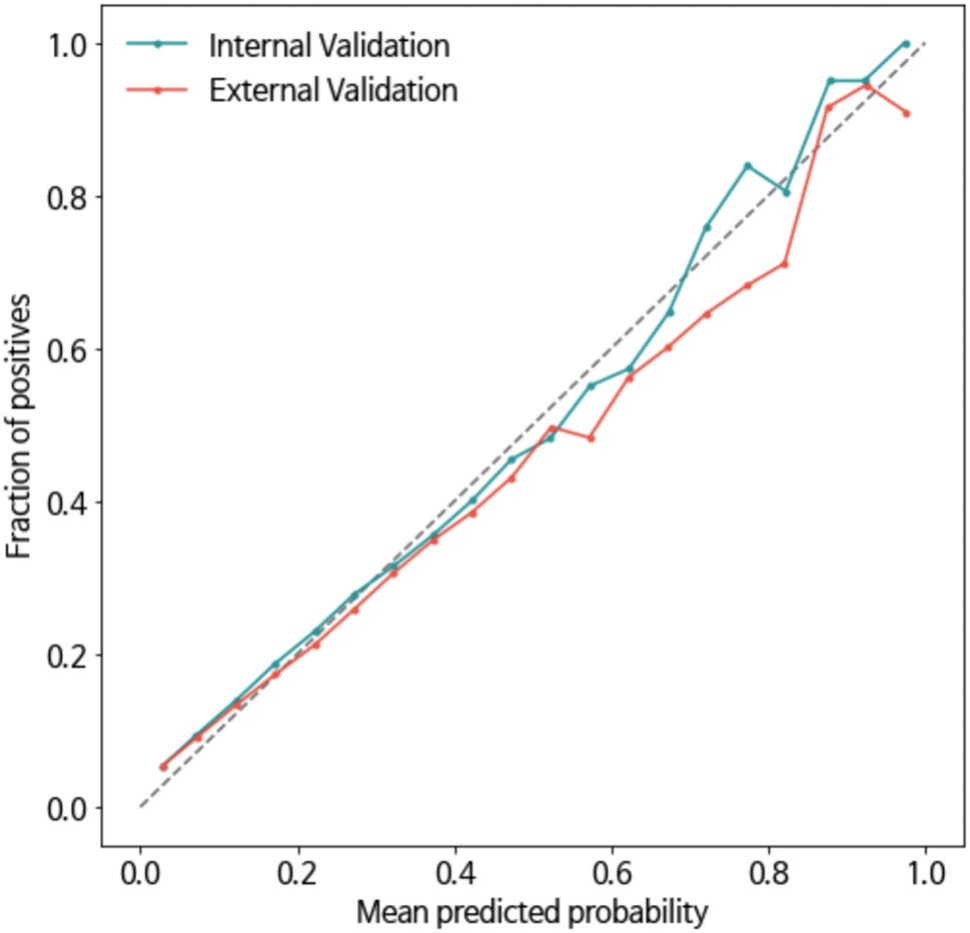
Cross-validated model calibration for internal and external validation.

Through SFS, predictors were selected from all 146 variables, and the final model incorporated 93 features, including six sociodemographic, 28 comorbidity, and 59 medication-related variables; 13 general, 13 DDI, and nine disease-specific PIMs; and 23 other medication classes. In the training set, the final model (AUROC: 0.690) showed a better performance than the full model (AUROC: 0.687). It also showed better performance than the baseline model (AUROC: 0.659) based on previous studies. The test set, similar to the training set, also showed that the AUROC of the final model (0.689) was the highest compared to that of the other variable groups, which meant that the final model performed outstanding classification and was robust (Fig. 3). The accuracy, sensitivity, specificity, PPV, NPV, and F1-score of the final model in the test set was 0.669, 0.598, 0.678, 0.189, 0.931, and 0.287, respectively, as shown in Table 2. In the training set, the McFadden Pseudo R-square of the logistic regression was 0.129.

**Table 2:** Comparison of predictive performance between each combination of the variable’s category model.

Data set	Category	AUROC	ACC	SEN	SPE	PPV	NPV	F1
Training set	Sociodemographic and comorbidity	0.656 (0.652, 0.660)	0.646 (0.624, 0.668)	0.571 (0.538, 0.603)	0.655 (0.626, 0.684)	0.172 (0.167, 0.176)	0.924 (0.922, 0.927)	0.264 (0.261, 0.266)
	Medication use	0.665 (0.661, 0.668)	0.680 (0.664, 0.697)	0.545 (0.524, 0.566)	0.697 (0.676, 0.718)	0.184 (0.179, 0.189)	0.925 (0.923, 0.926)	0.275 (0.271, 0.278)
	Baseline model	0.659 (0.655, 0.663)	0.664 (0.625, 0.704)	0.553 (0.499, 0.607)	0.678 (0.627, 0.729)	0.178 (0.167, 0.188)	0.924 (0.921, 0.927)	0.268 (0.263, 0.273)
	Full model	0.688 (0.685, 0.690)	0.685 (0.671, 0.699)	0.575 (0.560, 0.590)	0.699 (0.682, 0.717)	0.193 (0.188, 0.198)	0.929 (0.929, 0.930)	0.289 (0.285, 0.293)
	Final model	0.690 (0.687, 0.693)	0.670 (0.636, 0.704)	0.599 (0.557, 0.640)	0.679 (0.635, 0.722)	0.190 (0.179, 0.200)	0.931 (0.929, 0.934)	0.287 (0.280, 0.295)
Test set	Sociodemographic and comorbidity	0.651 (0.651, 0.651)	0.666 (0.646, 0.687)	0.536 (0.510, 0.562)	0.682 (0.656, 0.709)	0.174 (0.169, 0.179)	0.922 (0.920, 0.923)	0.263 (0.261, 0.265)
	Medication use	0.667 (0.667, 0.667)	0.656 (0.643, 0.669)	0.581 (0.564, 0.597)	0.665 (0.648, 0.682)	0.178 (0.175, 0.181)	0.927 (0.926, 0.928)	0.273 (0.271, 0.275)
	Baseline model	0.659 (0.659, 0.659)	0.664 (0.643, 0.684)	0.558 (0.532, 0.584)	0.677 (0.650, 0.703)	0.178 (0.172, 0.183)	0.925 (0.923, 0.926)	0.269 (0.266, 0.272)
	Full model	0.688 (0.688, 0.688)	0.663 (0.645, 0.681)	0.604 (0.580, 0.627)	0.671 (0.648, 0.694)	0.187 (0.182, 0.191)	0.931 (0.930, 0.933)	0.285 (0.282, 0.288)
	Final model	0.689 (0.689, 0.689)	0.669 (0.653, 0.685)	0.598 (0.578, 0.619)	0.678 (0.658, 0.699)	0.189 (0.184, 0.193)	0.931 (0.930, 0.932)	0.287 (0.284, 0.290)

The number in parentheses means a 95% confidence interval.

*AUROC* area under the receiver operating characteristics curve, *ACC* accuracy, *SEN* sensitivity, *SPE* specificity, *PPV* positive predictive value, *NPV* negative predictive value, *F1* F1-score.

### Model interpretation

**Figure 4:**
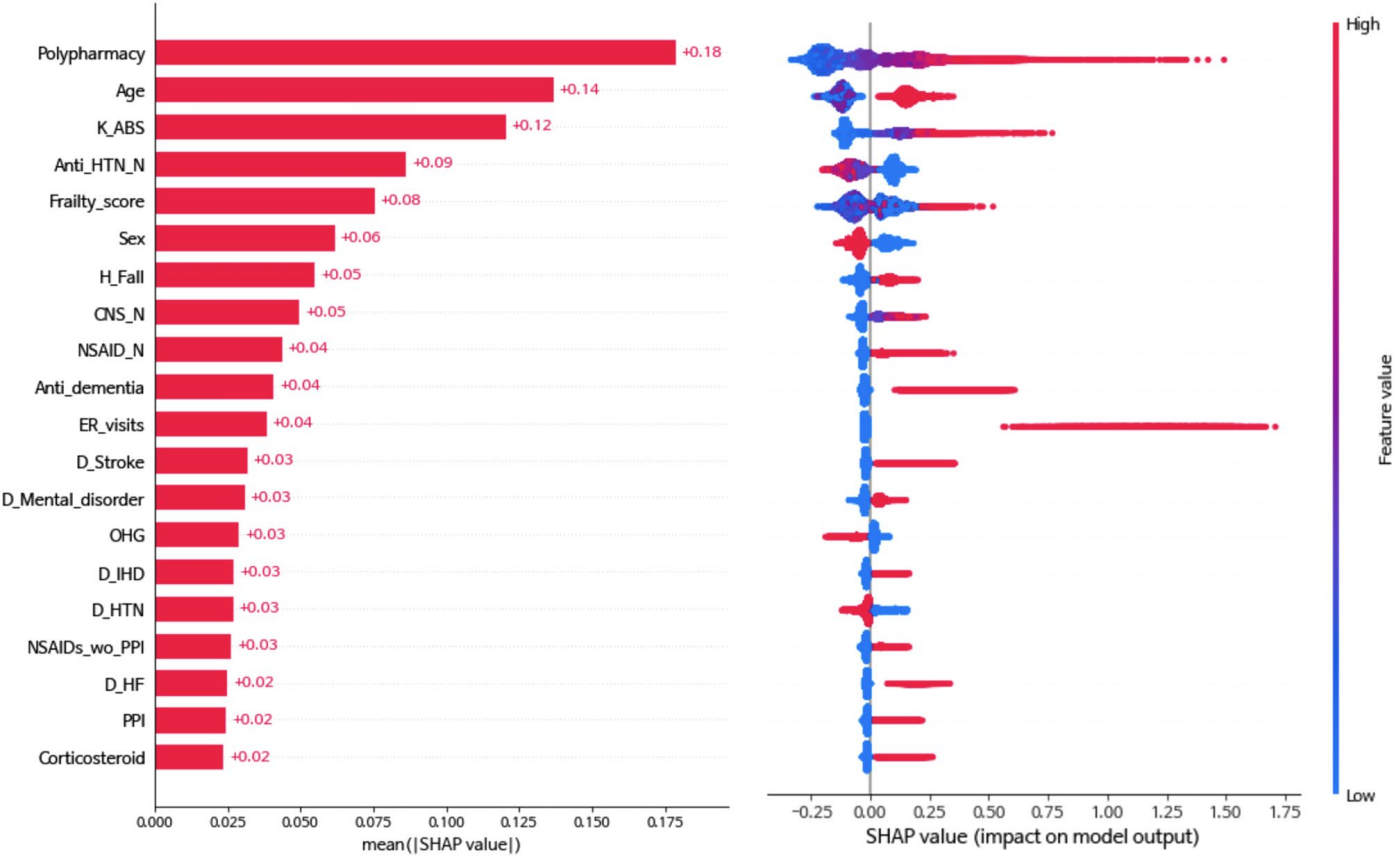
Quantification of feature impact on prediction through analysis of Shapley additive explanations (SHAP) values of the 20 most impactful features for the final model. *K_ABS* Korean-Anticholinergic burden scales, *Anti_HTN_N* number of antihypertensive agents, *H_Fall* history of fall or fractures, *CNS_N* number of central nervous system agents, *NSAID_N* number of non-steroidal anti-inflammatory agents, *Anti_dementia* antidementia agents, *D_strokes* stroke, *D_mental_disorder* mental disorder, *OHG* oral hyperglycemic agents, *D_IHD* ischemic heart disease, *D_HTN* hypertension, *NSAIDs_wo_PPI* non-steroidal anti-inflammatory agents without using proton pump inhibitors, *D_HF* heart failure, *PPI* proton pump inhibitor.

**Figure 5:**
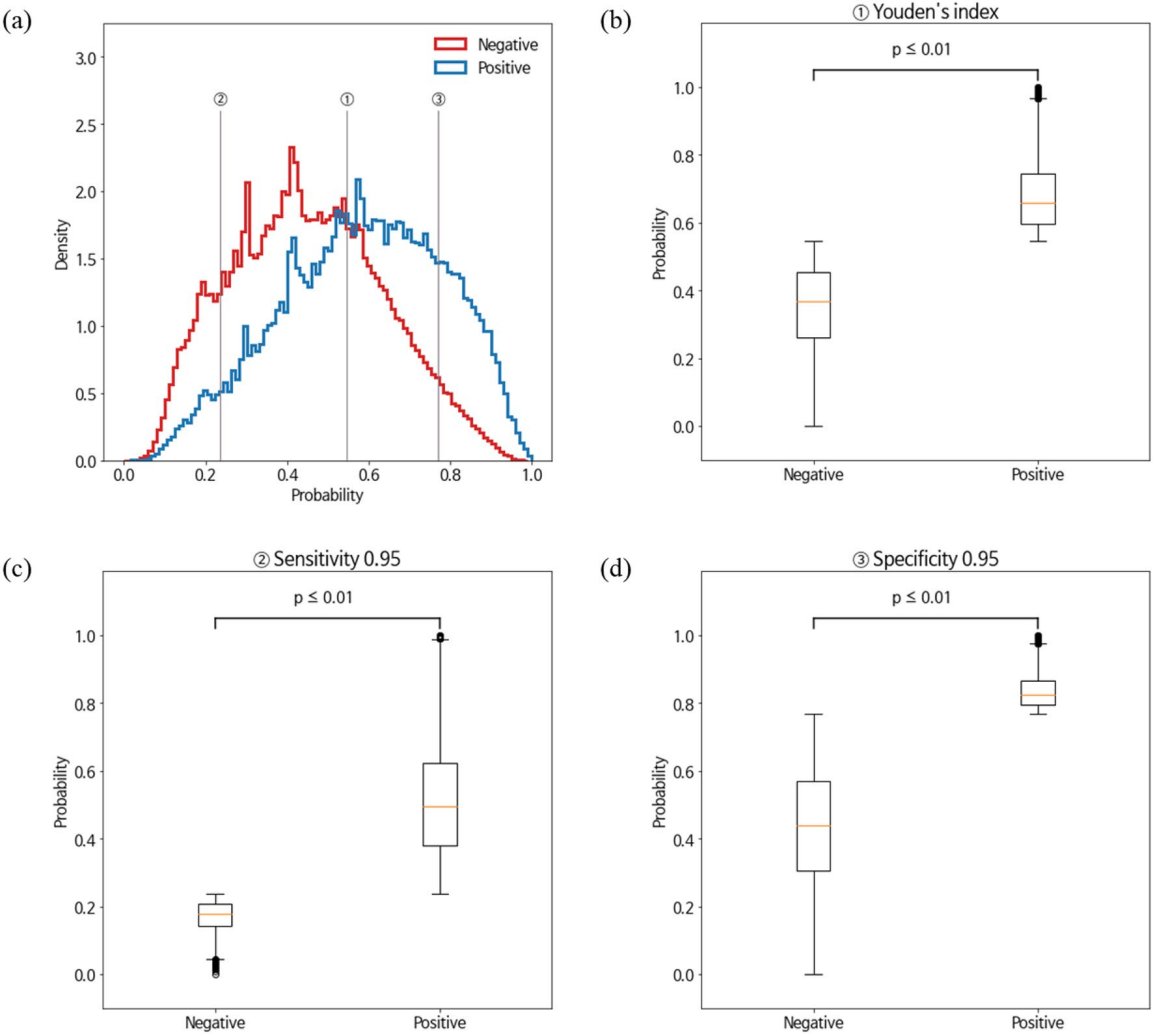
The predicted probability distribution of emergnecy department (ED) visit (blue line) and non-ED visit (red line) (**a**). The distribution classified through cut-off probability is specified as follows: (**b**) optimal point by Youden's index (**c**) sensitivity 0.95 (**d**) specificity 0.95.

SHAP values were used to show how the LightGBM model classified ED visits using the variables of the present study. One of the five repeated validation datasets (training set) was randomly selected and demonstrated. The top 20 SHAP values for the variable categories and the full model are shown in Supplementary Fig. 1. In the final model, the top 10 features with the highest predictive power among the 93 features incorporated were polypharmacy, age group, K-ABS score, number of antihypertensive drugs, frailty score, sex, history of falls or fractures, number of central nervous system agents, non-steroidal anti-inflammatory drugs, and antidementia agents (Fig. 4). The variable importance of the other models, including the baseline model and the full model, is presented in Supplementary Fig. 2. The mean absolute SHAP values of these top three variables exceeded 0.1, which tended to be positively correlated with ED visits in older patients. On the other hand, the fourth most impactful variable, the number of antihypertensive drugs, tended to be negatively correlated with ED visits. Table 3 shows the classification performance of the final model for each cutoff probability. In addition, Fig. 5 shows that when using Youden’s index with 95% sensitivity and 95% specificity as the threshold, the distribution of ED and non-ED visitors was significantly different (Mann–Whitney U test: *P* ≤ 0.01).

**Table 3:** Classification performance of the final model for different cut-off probability.

Cut-off	TP	TN	FP	FN	ACC	SEN	SPE	PPV	NPV	F1
0.0	54,274	0	434,192	0	0.111	1.000	0.000	0.111	0.000	0.200
0.1	54,124	3776	430,416	150	0.119	0.997	0.009	0.112	0.962	0.201
0.2	52,508	42,779	391,413	1766	0.195	0.967	0.099	0.118	0.960	0.211
0.3	49,282	103,757	330,435	4992	0.313	0.908	0.239	0.130	0.954	0.227
0.4	43,999	178,652	255,540	10,275	0.456	0.811	0.411	0.147	0.946	0.249
0.5	36,097	262,562	171,630	18,177	0.611	0.665	0.605	0.174	0.935	0.276
0.6	26,387	338,317	95,875	27,887	0.747	0.486	0.779	0.216	0.924	0.299
0.7	16,961	389,913	44,279	37,313	0.833	0.313	0.898	0.277	0.913	0.294
0.8	8484	419,590	14,602	45,790	0.876	0.156	0.966	0.367	0.902	0.219
0.9	2062	432,403	1789	52,212	0.889	0.038	0.996	0.535	0.892	0.071
1.0	1	434,192	0	54,273	0.889	0.000	1.000	1.000	0.889	0.000

*TP* true positive, *TN* true negative, *FP* false positive, *FN* false negative, *ACC* accuracy, *SEN* sensitivity, *SPE* specificity, *PPV* positive predictive value, *NPV* negative predictive value, *F1* F1-score.

### Model performance in subgroup

The model performance remained largely consistent across subgroups (Supplementary Table 6). However, discrimination was relatively lower for patients aged 65–69 and for patients with a history of ED visits in the previous 3 months, both in the training set and the test set. For instance, in the training set, the model achieved a discrimination score of 0.655 for patients aged 65–69, 0.688 for those aged 70–74, and 0.684 for patients aged 75 years or older.

## Discussion

Here, an ML-based model was developed and validated that predicted the risk of ED visits in older patients with comprehensive medication-related variables using nationwide claims data. The final model comprised 93 predictors, including 34 sociodemographic and comorbidity variables as well as 59 medication-related variables.

Contrary to expectations, the final model of the present study demonstrated lower performance compared to a previous study by Hippisley and Coupland^7^, which focused on the development of a model for predicting emergency admission (AUROC: 0.78). Owing to differences in prescription patterns and healthcare systems by country, it is difficult to generalize existing risk prediction models to other populations. Similarly, when applying Hippisley and Coupland’s model (baseline model)^7^ to the population in the present study, it showed lower performance (AUROC: 0.659) than what the authors had suggested (AUROC: 0.78). Several factors accounted for this discrepancy. First, Hippisley and Coupland included all patients aged 18–100 years old and developed the model using the QResearch database, which contains health records of patients registered from general practices. In contrast, the present study included elderly individuals and excluded patients with cancer who had specific characteristics such as fever, malnutrition, neutropenia, and post-surgical complications, as they differed from other non-cancer patients^32^. Additionally, the claims data used in this study did not include variables such as ethnicity, smoking, alcohol status, and laboratory test results, which were present in Hippisley and Coupland’s model. However, to compensate for the unavailability of these features in the claims data, we utilized comprehensive medication data.

In line with previous study, polypharmacy, anticholinergics, NSAIDs, history of ED visit, ischemic heart disease, central nervous system agents, were remarkable factors according to its top 20 important SHAP values^33,34^. Also, the final model of this study included most of the variables in Hippisley and Coupland’s final model, which was developed using a logistic regression method. Among the available variables, venous thromboembolism (VT) was excluded from the final model. Instead, anticoagulants, including both oral and IV formulations, which are commonly used to treat VT, were included as strong predictors in the final model. In addition, a Scottish study that predicted emergency hospitalization with drugs used over the past 3 years showed similar results to those of the present study in that antibacterial agents and diuretics were strong predictors^8^. However, unlike the present study, analgesics, including opioids, were also included in the final model as strong predictors. This can be explained by the fact that patients with cancer who chronically use opioids were included in their study.

Among the ML models, GBM-based models (XGBoost, LightGBM, CatBoost) are state-of-the-art boosting models that show the best performance for a general classification problem^35–37^. In this study, it was also seen that the AUROC of the GBM-based model was higher than that of the other models. These results are attributed to the learning method of the GBM-based model, which learns several weak learners sequentially and proceeds with learning in a way that improves errors by weighting incorrectly predicted data^31^. Among the three GBM-based models, LightGBM's AUROC was the highest, which was possible because, compared to the other two models, LightGBM uses a different tree splitting method. Most existing tree-based algorithms (including XGBoost and CatBoost) use the level-wise tree split method to effectively reduce the tree depth. The reason for creating a balanced tree is that it has a more robust structure for overfitting. In contrast, LightGBM uses a leaf-wise tree split method to divide the leaf node with maximum delta loss, deepening the depth of the tree and creating an asymmetric tree. Leaf-centered tree segmentation is said to be easy to overfit for a small amount of data. However, because a large amount of data was used in this study, LightGBM could perform best by taking advantage of the leaf-centered tree segmentation method while avoiding overfitting^38^. In the category of variable analysis, which was experimented with by changing variable groups using LightGBM, the AUROC of final models using 93 variables was higher than that of other variable groups or full models using 146 variables. In contrast to the full model, the final model used useful variables that removed useless variables through the SFS; thus, the final model could obtain the best performance.

To the best of our knowledge, this is the first study to predict ED visits among community-dwelling older noncancer patients using ML approaches. One of the main strengths of this study is that it used comprehensive medication-related variables, sociodemographics, and comorbidities, which are easy to apply in the field. However, this study does possess limitations attributed to its relatively low predictability, despite the utilization of diverse machine learning and AI models. Firstly, constraints within the claims data prevented the inclusion of more precise clinical indicators such as laboratory test results, which could offer a better representation of each patient's condition. As a result, our model exhibited suboptimal predictive capability compared to existing models. Secondly, our assumption that each patient had fully adhered to prescribed medications, excluding over-the-counter drugs, might have underestimated the influence of certain medications on the likelihood of an ED visit. Thirdly, due to the rarity of the outcome (ED visits) and the limited data duration, a case–control design was adopted, preventing the determination of ED visit incidence rates among older patients. Additionally, predictions were based on information from the month immediately preceding the ED visit occurrence. Fourthly, the issue of diagnosis code accuracy in claims data persisted. Nevertheless, our model’s performance using only administrative data was satisfactory in both derivation and test sets, indicating its potential utility in identifying older adults likely to require an ED visit on a nationwide scale. While our model's predictive capability fell short of initial expectations, its value remains significant due to its derivation from nationwide claims data, potentially offering insight into the entire Korean population. Its automated data acquisition feature is pivotal in identifying high-risk groups for ED visits without necessitating additional assessments. This presents an opportunity for integration into a nationwide prospective drug utilization review program, enabling targeted preventive interventions for medication-related ED visits.

Owing to the limitations of nationwide claims data, the prediction model developed with only claims data might be suboptimal compared to those developed with combined data, including those obtained from patient interviews or clinical records. However, one goal of the present analysis was to create a prediction model with the variables that could be obtained from claims data that are expected to be widely applied because of their easily computable and identifiable properties. This model enables nationwide screening of elderly patients who are likely to require a visit to an ED and supports the selection of target populations for managing high-risk elderly individuals. Nevertheless, it is worth considering further approaches to enhance the predictive ability of the model, such as combining LightGBM with other neural network models or incorporating other datasets with claims data to compensate for the shortcoming of claims data.

## Conclusion

In this study, an ED visit risk prediction model for community-dwelling older patients was developed and validated using administrative data, and it was found that medication-related variables were helpful for predicting the likelihood of an ED visit. Implementation of the final model in a clinical setting could contribute to the risk stratification of older patients who are likely to require ED visits, and ultimately reduce the socioeconomic burden for both patients and the healthcare system.

### Supplementary Information

Supplementary Information.

## Data Availability

The data presented in this study are available on request from the corresponding author.
