# Supplementary material for: Machine learning-based prediction model for emergency department visits using prescription information in community-dwelling non-cancer older adults
Source: Sci Rep. 2023 Nov 2;13:18887. doi: 10.1038/s41598-023-46094-z (PMC10622449; doi:10.1038/s41598-023-46094-z)
Supplement: Supplementary file 1 — Supplementary Information. [file 41598_2023_46094_MOESM1_ESM.docx]

**Supplementary table 1. List of predictors considered in our model (143 variables)**

| **Domain** | **Predictors** |
| --- | --- |
| **Sociodemographic and comorbidity (38 predictors)** | |
| Sociodemographic factors (3 predictors) | Age*, insurance status*, sex* |
| Healthcare Utilization pattern (2 predictors) | History of emergency department visit*, history of hospitalization* |
| Frailty  (1 predictor) | Frailty score* |
| Comorbidity (32 predictors) | Parkinson's disease*, cognitive impairment*, hypertension*,  arrhythmia*, ischemic heart disease*, myocardial infarction*, stroke*, plegia*, heart failure, diabetes*, COPD, asthma*, mild to moderate liver disease*, moderate liver disease*, mild to severe renal disease*, severe renal disease*, hyperkalemia*, hypokalemia*, hyponatremia*, pneumonia*, anemia*, urinary retention*, syncope or bradycardia or hypotension*, major bleeding*, GI bleeding*, peptic ulcer disease*, mental disorder*, history of fall or fractures*, rheumatic arthritis, gout, deep vein thrombosis or pulmonary embolism, peripheral vascular disease |
| **Medication-related predictors (108 predictors)** | |
| Medication class (34 predictors) | Antidepressants*, selective serotonin reuptake inhibitors*, sedative-hypnotic drugs*, beta-blockers*, non-dihydropyridine calcium channel blockers, oral hyperglycemic agents*, insulin*, anticoagulant*, P2Y12 inhibitors*, acetylsalicylic acid*, antiepileptic drugs (except for gabapentinoid), corticosteroid*, H2 receptor antagonists*, proton-pump inhibitors*, immunosuppressants*, oral antineoplastic drug*, COX2 inhibitor*, anti-dementia drug*, thiazide diuretics*, potassium-sparing diuretics*, methotrexate*, antimuscarinic drugs for urinary incontinence*, aminoglycosides*, antiparkinson agent, serotonin and norepinephrine reuptake inhibitors, antihypertensive drug, oral anticoagulants, gabapentinoid, antiarrhythmic drugs, bisphosphonate, tramadol, opioids (except for tramadol), acetylcholinesterase inhibitor, loop diuretics |
| General PIMs (19 predictors) | Anticholinergic antiparkinson agents*, tricyclic antidepressant, antipsychotic*, Z-drugs*, long-acting sulfonylurea*, non-steroidal anti-inflammatory agents without using proton pump inhibitors*, anticholinergic skeletal muscle relaxants*, digoxin*, antispasmodic*, peripheral alpha-1 blockers*, desmopressin*, megestrol*, metoclopramide*, typical antipsychotics, long-acting benzodiazepines, estrogen or testosterone, first-generation antihistamine, theophylline, barbiturates |
| DDI PIMs (30 predictors) | Polypharmacy*, aldosterone antagonists and potassium-sparing drugs*, opioids and gabapentinoid, opioids and benzodiazepines, lithium and RAS inhibitor, lithium and loop diuretics, peripheral α-1 blockers and loop diuretics, phenytoin and trimethoprim-sulfamethoxazole, Theophylline and Cimetidine, Theophylline and Ciprofloxacin, Warfarin and Amiodarone, Warfarin and Ciprofloxacin, Warfarin and Macrolides (except for azithromycin), Warfarin and Trimethoprim-Sulfamethoxazole, Anticoagulant (oral) and tNSAIDs*, Beta blocker in combination with verapamil or diltiazem, Acetylcholinesterase inhibitor and beta-blockers, digoxin, diltiazem or verapamil, Use of regular opioids without concomitant laxative, Bisphosphonates and vitamin D and calcium in patient taking long-term systemic corticosteroid therapy, Folic acid supplement in patient taking methotrexate/ Methotrexate without using folic acid, RAS inhibitor and NSAIDs, Anticoagulant and Antiplatelet, Korean-Anticholinergic burden scales*, Number of used strong anticholinergic drugs concomitantly*, Number of used Antihypertensive agents concomitantly, * Number of used Benzodiazepines concomitantly*, Number of used CNS-active agents concomitantly*, Number of used Loop diuretics concomitantly*, Number of used NSAIDs concomitantly*, Number of used RAS inhibitors concomitantly* |
| Disease-specific PIMs (25 predictors) | - Peripheral alpha-1 blockers for treatment of hypertension* - NSAIDs and COX-2 inhibitors in patients with heart failure* Anticholinergics in patients with dementia or cognitive impairment* - CNS active drugs in patients with history of falls or fractures* - Antipsychotics in patients with Parkinson disease (Except quetiapine, clozapine) * - NSAIDs in patients with history of gastric or duodenal ulcers* - NSAIDs in patients with chronic kidney disease stage 4 or higher* - Strongly anticholinergic drugs for urinary incontinence in women with   lower urinary tract symptoms or benign prostatic hyperplasia*   - ACE inhibitors or Angiotensin receptor blockers in patients with hyperkalemia* - Nondihydropyridine CCBs in patients with heart failure - Thiazolidinediones in patients with heart failure - Cilostazole in patients with heart failure - Dronedarone in patients with heart failure - AchEI in patients with syncope - Benzodiazepines in patients with dementia or cognitive impairment - Z-drugs in patients with dementia or cognitive impairment, - Antipsychotics in patients with dementia or cognitive impairment - Antiemetics in patients with Parkinson disease - Estrogen (oral, transdermal) in women with urinary incontinence - Peripheral alpha-1 blockers in women with urinary incontinence - Betablocker in patients with bradycardia - Thiazide in patients with history of gout - Antimuscarinics drugs or tricyclic antidepressants with a history of narrow angle glaucoma - AchEI in patients with a known history of persistent bradycardia - Drugs likely to cause constipation in patients with chronic constipation where non-constipating alternatives are available |
|  |  |
|  |  |

* Predictors included in the optimized model (93 variables)

**Supplementary Table 2. Comparison of predictive performance between machine learning models with all variables**

| **Model** | **AUC** | **ACC** | **SEN** | **SPE** | **PPV** | **NPV** | **p-value** |
| --- | --- | --- | --- | --- | --- | --- | --- |
| LightGBM | 0.688 | 0.685 | 0.575 | 0.699 | 0.193 | 0.929 | Reference |
| Logistic Regression | 0.672 | 0.674 | 0.571 | 0.686 | 0.186 | 0.928 | < 0.001 |
| Linear Discriminant Analysis | 0.681 | 0.686 | 0.566 | 0.701 | 0.192 | 0.928 | < 0.001 |
| Random Forest | 0.658 | 0.651 | 0.576 | 0.660 | 0.175 | 0.926 | < 0.001 |
| XGBoost | 0.685 | 0.668 | 0.593 | 0.678 | 0.187 | 0.930 | < 0.001 |
| CatBoost | 0.687 | 0.663 | 0.602 | 0.670 | 0.186 | 0.931 | 0.006 |
| Deep Neural Network | 0.685 | 0.666 | 0.593 | 0.675 | 0.186 | 0.930 | < 0.001 |
| TabNet | 0.685 | 0.674 | 0.587 | 0.685 | 0.189 | 0.930 | < 0.001 |

AUC: Area under the receiver operating characteristics curve; ACC: Accuracy; SEN: Sensitivity; SPE: Specificity; PPV: Positive predictive value; NPV: Negative predictive value

**Supplementary Table 3. Hyperparameter for each model in the grid search**

| **Model** | **Range of the hyperparameter** | **Optimal hyperparameter** |
| --- | --- | --- |
| Logistic Regression | max_iter: 100, 250, 500, 750, 1000  C: 0.75, 0.8, 0.85, 0.9, 0.95, 1.0  tol: 0.0001, 0.0005, 0.001, 0.005, 0.001 | max_iter: 250  C: 0.9  tol: 0.001 |
| Linear Discriminant Analysis | tol: 0.0001, 0.0005, 0.001, 0.005, 0.001 | tol: 0.0005 |
| Random Forest | n_estimators: 100, 250, 500, 750, 1000  max_depth: 5, 10, 15, 20, 25, 50 | n_estimators: 100  max_depth: 15 |
| XGBoost | n_estimators: 100, 250, 500, 750, 1000  learning_rate: 0.001, 0.005, 0.01, 0.05, 0.1  max_depth: 5, 10, 15, 20, 25, 50 | n_estimators: 750  learning_rate: 0.001  max_depth: 25 |
| LightGBM |  | n_estimators: 1000  learning_rate: 0.005  max_depth: 10 |
| CatBoost |  | n_estimators: 500  learning_rate: 0.01  max_depth: 25 |
| Deep Neural Network | number of hidden layers: 5, 6, 7, 8, 9, 10  activation: logistic, tanh, relu  optimizer: lbfgs, sgd, adam  learning_rate: 0.0001, 0.0005, 0.001, 0.005, 0.01  max_iter: 100, 250, 500, 750, 1000 | number of hidden layers: 5  activation: relu  optimizer: adam  learning_rate: 0.0005  max_iter: 500 |
| TabNet | learning_rate: 0.001, 0.005, 0.01, 0.05, 0.1  n_d: 8, 16, 24, 32, 40, 48, 56, 64  n_a: 8, 16, 24, 32, 40, 48, 56, 64  n_steps: 3, 4, 5, 6, 7, 8, 9, 10  optimizer: sgd, adam  max_epochs: 100, 250, 500, 750, 1000 | learning_rate: 0.005  n_d: 24  n_a: 24  n_steps: 5  optimizer: adam  max_epochs: 250 |

**Supplementary Table 4. Hyperparameter for the combination of the variable’s category model in the grid search**

| **Category** | **Range of the hyperparameter** | **Optimal hyperparameter** |
| --- | --- | --- |
| Sociodemographic and comorbidity | n_estimators: 100, 250, 500, 750, 1000  learning_rate: 0.001, 0.005, 0.01, 0.05, 0.1  max_depth: 5, 10, 15, 20, 25, 50 | n_estimators: 250  learning_rate: 0.01  max_depth: 10 |
| Medication use |  | n_estimators: 250  learning_rate: 0.01  max_depth: 15 |
| Baseline model |  | n_estimators: 500  learning_rate: 0.001  max_depth: 10 |
| Full model |  | n_estimators: 1000  learning_rate: 0.005  max_depth: 15 |
| Optimized model |  | n_estimators: 750  learning_rate: 0.005  max_depth: 20 |

**Supplementary table 5. Patient’s characteristics of the training set and test set**

| **Variables** | **Training set** | | | | | | **Test set** | | | |
| --- | --- | --- | --- | --- | --- | --- | --- | --- | --- | --- |
|  | **Case  (N=54,274)** | | **Control (N=434,192)** | | **p-value** | **AUC** | **Case  (N=56,762)** | **Control (N=454,096)** | **p-value** | **AUC** |
| **Sociodemographic** |  | | | | | |  |  |  |  |
| **Age** |  |  | |  | | 0.559 |  |  |  | 0.573 |
| 65~69 | 14041 (25.9) | | 143997 (33.2) | | <0.001 |  | 14301 (25.2) | 148681 (32.7) | <0.001 |  |
| 70~74 | 11754 (21.7) | | 110681 (25.5) | |  |  | 12146 (21.4) | 116562 (25.7) |  |  |
| ≥75 | 28479 (52.5) | | 179514 (41.3) | |  |  | 30315 (53.4) | 188853 (41.6) |  |  |
| **Insurance status** |  |  | |  | | 0.519 |  |  |  | 0.521 |
| Health insurance | 47893 (88.2) | | 399080 (91.9) | | <0.001 |  | 49883 (87.9) | 418348 (92.1) | <0.001 |  |
| Medical aid | 6381 (11.8) | | 35112 (8.1) | |  |  | 6879 (12.1) | 35748 (7.9) |  |  |
| **Sex** |  | |  | |  | 0.490 |  |  |  | 0.490 |
| Male | 23102 (42.6) | | 176448 (40.6) | | <0.001 |  | 24314 (42.8) | 185184 (40.8) | <0.001 |  |
| **Frailty score** |  | |  | |  | 0.580 |  |  |  | 0.570 |
| <5 | 40741 (75.1) | | 373195 (86.0) | | <0.001 |  | 46639 (82.2) | 408695 (90.0) | <0.001 |  |
| 5~≤10 | 8891 (16.6) | | 45887 (10.6) | |  |  | 8588 (15.1) | 41308 (9.1) |  |  |
| >10 | 4542 (8.4) | | 15110 (3.5) | |  |  | 1535 (2.7) | 4092 (0.9) |  |  |
| ER visits (previous 3 months) | 2895 (5.3) | | 5007 (1.2) | | <0.001 | 0.521 | 3098 (5.5) | 5264 (1.2) | <0.001 | 0.521 |
| Hospitalization (previous 3 months) | 5327 (9.8) | | 15212 (3.5) | | <0.001 | 0.532 | 5273 (9.3) | 16195 (3.6) | <0.001 | 0.500 |
| **Comorbidity** |  | |  | |  |  |  |  |  |  |
| Hypertension | 37106 (68.4) | | 284656 (65.6) | | <0.001 | 0.514 | 39019 (68.7) | 298387 (65.7) | <0.001 | 0.515 |
| Mental disorder | 27317 (50.3) | | 159616 (36.8) | | <0.001 | 0.568 | 28781 (50.7) | 169487 (37.3) | <0.001 | 0.567 |
| Diabetes | 21050 (38.8) | | 145211 (33.4) | | <0.001 | 0.527 | 22680 (40) | 156737 (34.5) | <0.001 | 0.527 |
| COPD | 16368 (30.2) | | 97710 (22.5) | | <0.001 | 0.538 | 16625 (29.3) | 98216 (21.6) | <0.001 | 0.538 |
| Peptic ulcer disease | 14417 (26.6) | | 93642 (21.6) | | <0.001 | 0.525 | 14764 (26) | 94233 (20.8) | <0.001 | 0.526 |
| Liver disease | 12562 (23.2) | | 80688 (18.6) | | <0.001 | 0.523 | 13743 (24.2) | 90968 (20) | <0.001 | 0.521 |
| Ischemic heart disease | 11314 (20.9) | | 61264 (14.1) | | <0.001 | 0.534 | 12044 (21.2) | 64171 (14.1) | <0.001 | 0.535 |
| Asthma | 9963 (18.4) | | 60757 (14) | | <0.001 | 0.522 | 10066 (17.7) | 59978 (13.2) | <0.001 | 0.523 |
| Stroke | 7484 (13.8) | | 37295 (8.6) | | <0.001 | 0.526 | 7926 (14) | 39252 (8.6) | <0.001 | 0.527 |
| Anemia | 6000 (11.1) | | 28298 (6.5) | | <0.001 | 0.523 | 6696 (11.8) | 31457 (6.9) | <0.001 | 0.524 |

*AUC: Area under the receiver operating characteristics curve; ED, Emergency department*

**Supplementary Table 6. Subgroup-specific AUROC of the final model in the training set and the test set**

| **Variables** | **Training set** | **Test set** |
| --- | --- | --- |
| **Age** |  |  |
| 65~69 | 0.665 | 0.664 |
| 70~74 | 0.688 | 0.686 |
| ≥75 | 0.684 | 0.691 |
| **Insurance status** |  |  |
| Health insurance | 0.687 | 0.686 |
| Medical aid | 0.687 | 0.690 |
| **Sex** |  |  |
| Male | 0.690 | 0.689 |
| Female | 0.689 | 0.690 |
| **Frailty score** |  |  |
| <5 | 0.674 | 0.678 |
| 5~≤10 | 0.690 | 0.690 |
| >10 | 0.685 | 0.689 |
| **ED visits (previous 3 months)** | 0.667 | 0.670 |
| **Hospitalization (previous 3 months)** | 0.709 | 0.705 |
| **Comorbidity** |  |  |
| Hypertension | 0.701 | 0.702 |
| Mental disorder | 0.688 | 0.688 |
| Diabetes | 0.702 | 0.703 |
| COPD | 0.700 | 0.704 |
| Peptic ulcer disease | 0.694 | 0.700 |
| Liver disease | 0.705 | 0.707 |
| Ischemic heart disease | 0.690 | 0.697 |
| Asthma | 0.705 | 0.708 |
| Stroke | 0.688 | 0.688 |
| Anemia | 0.715 | 0.716 |

*AUC: Area under the receiver operating characteristics curve; ED, Emergency department; COPD, Chronic obstructive pulmonary disease;*

(a)


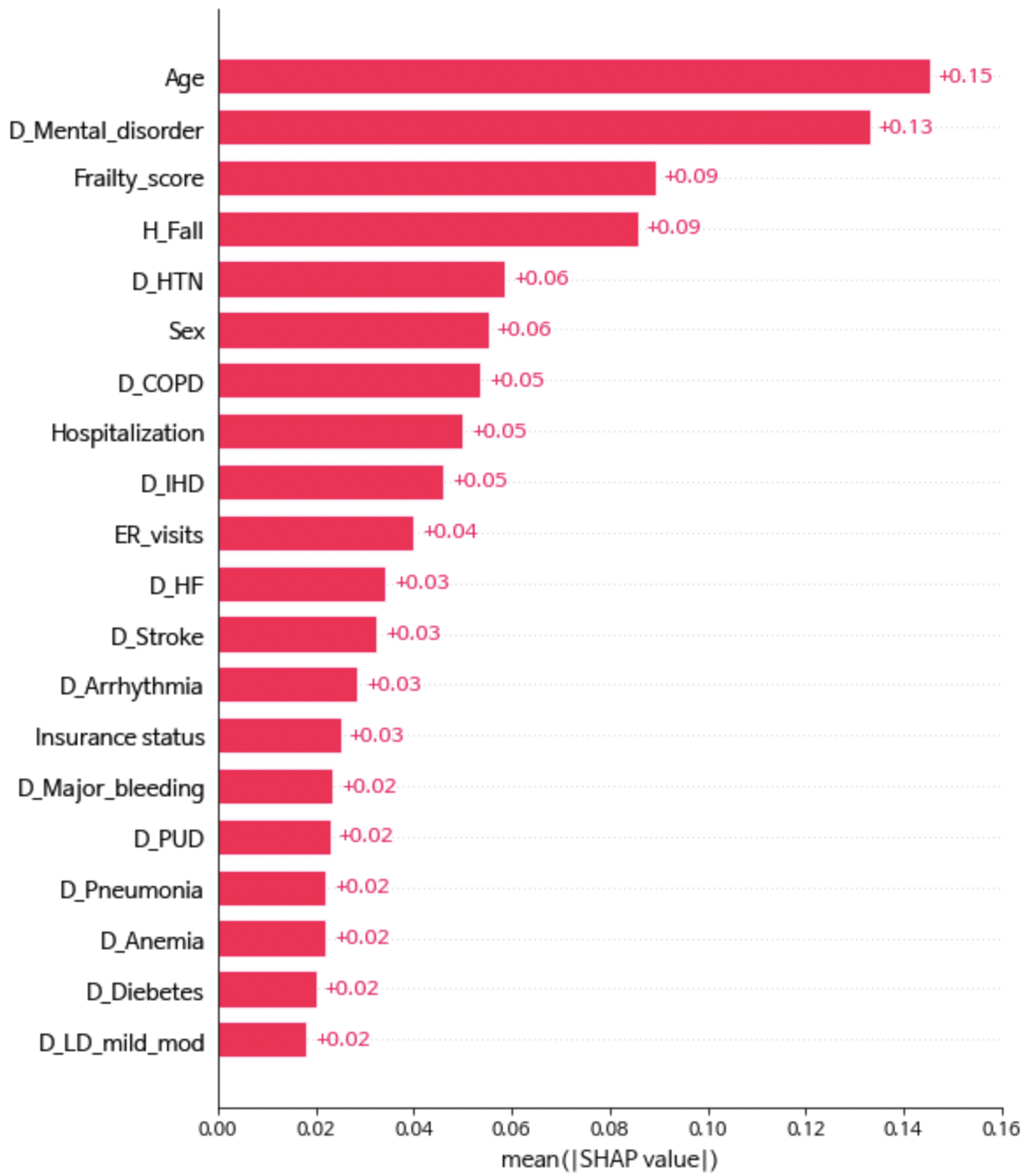

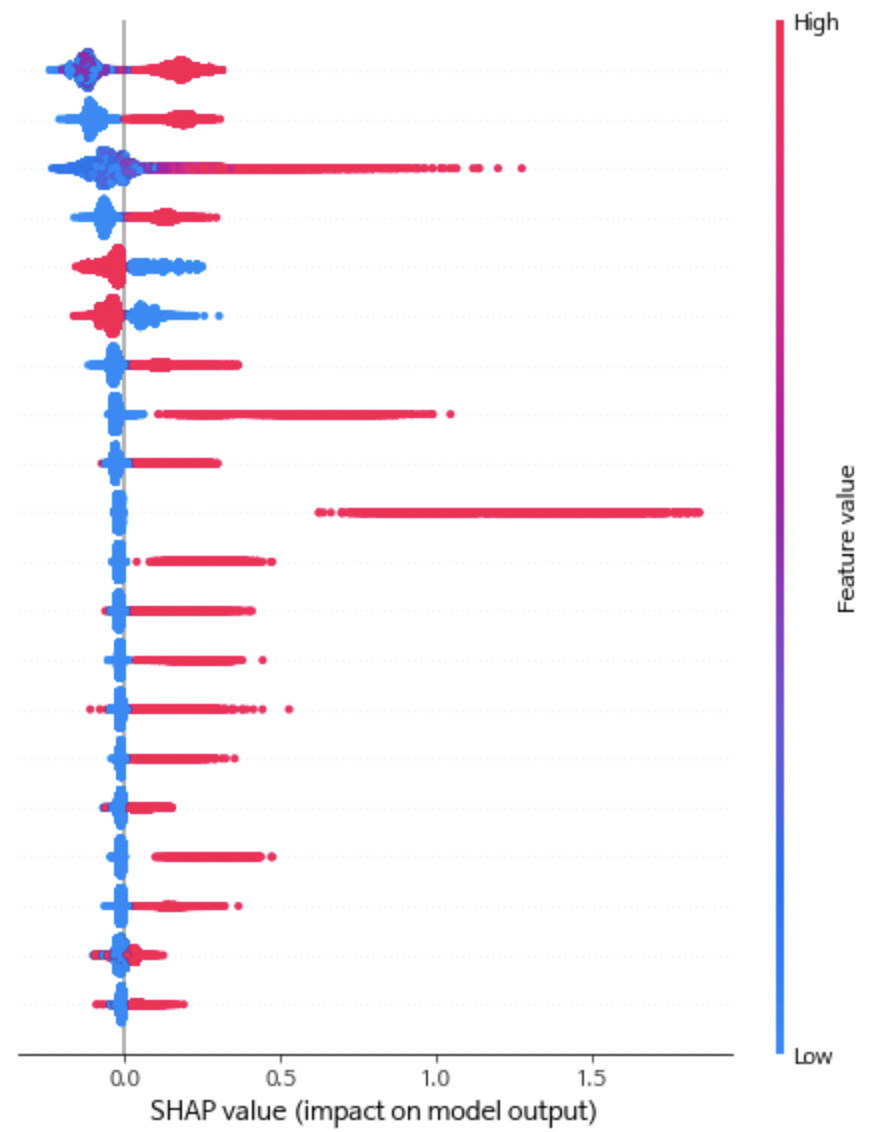


**(b)**


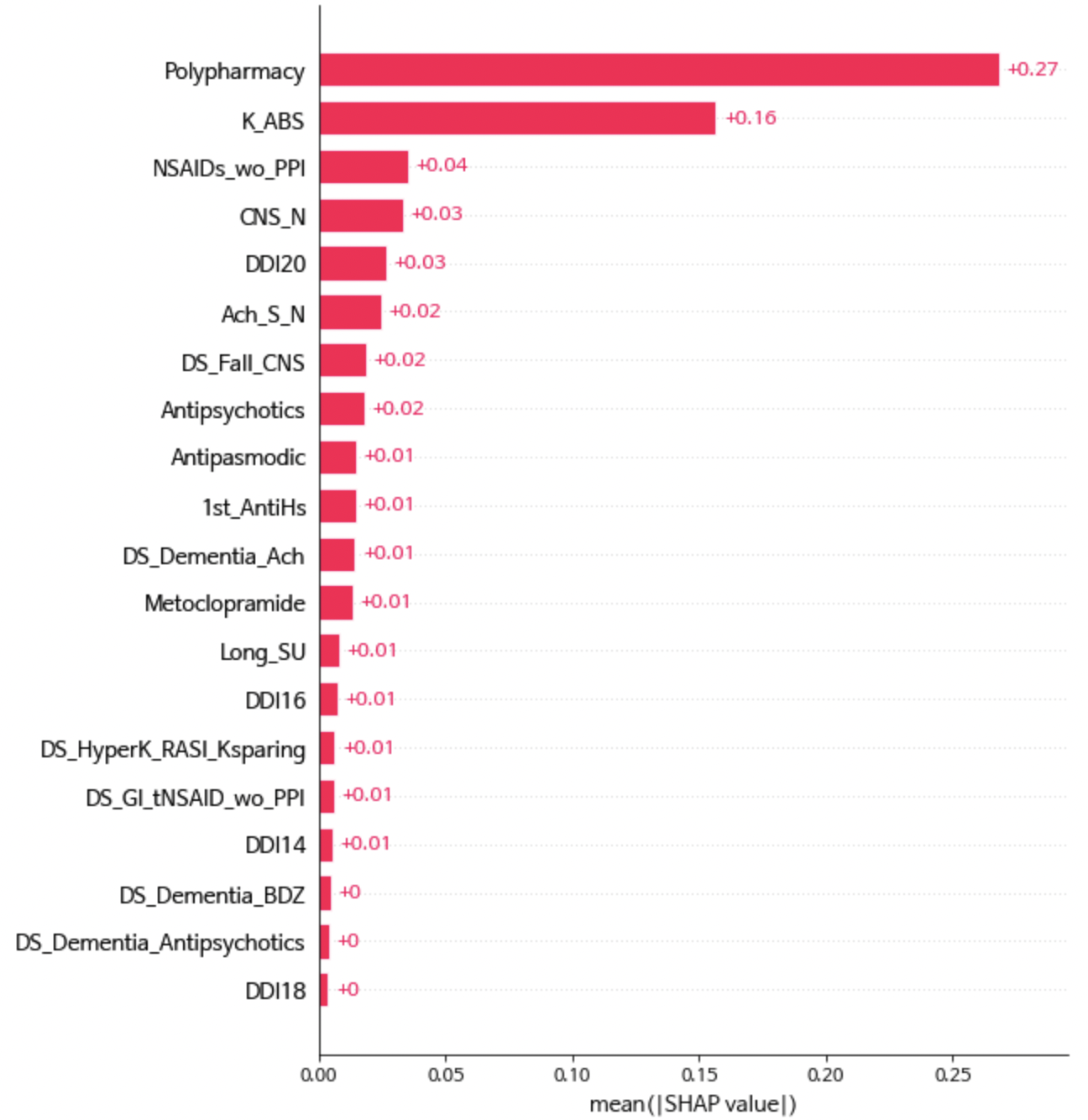

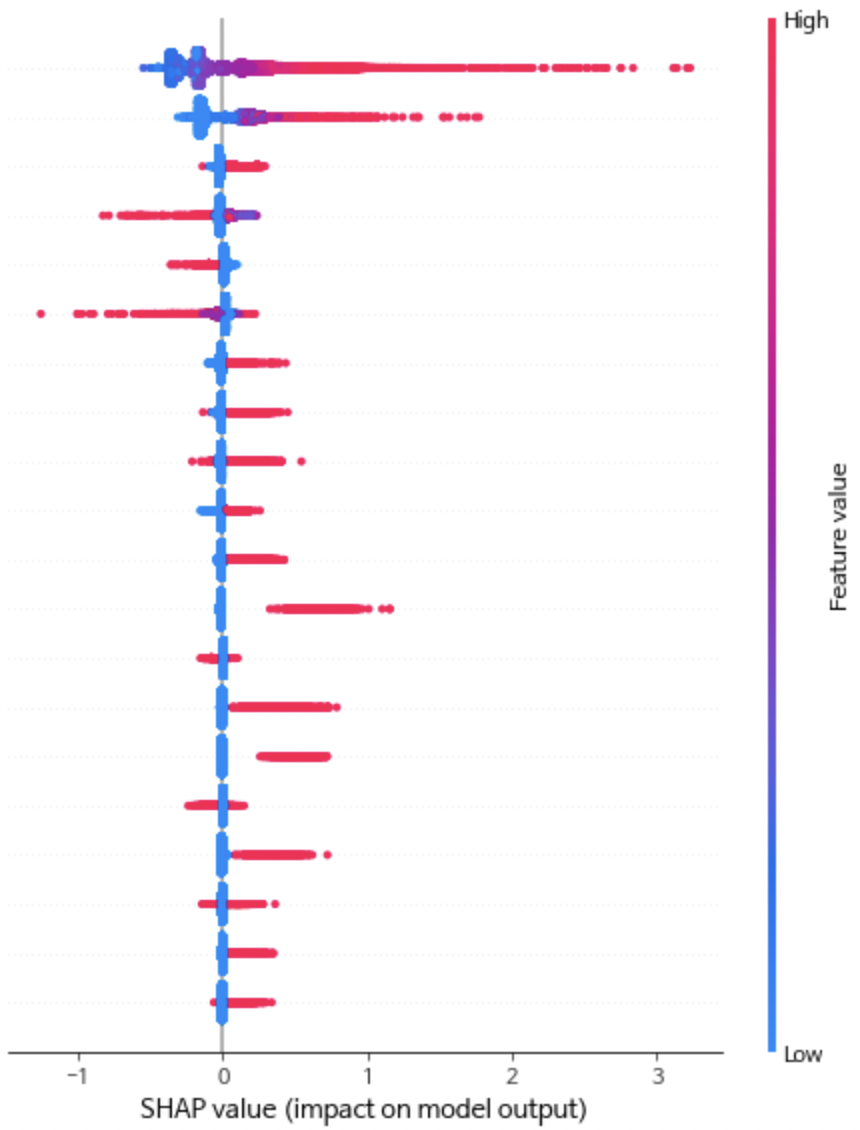


**(c)**


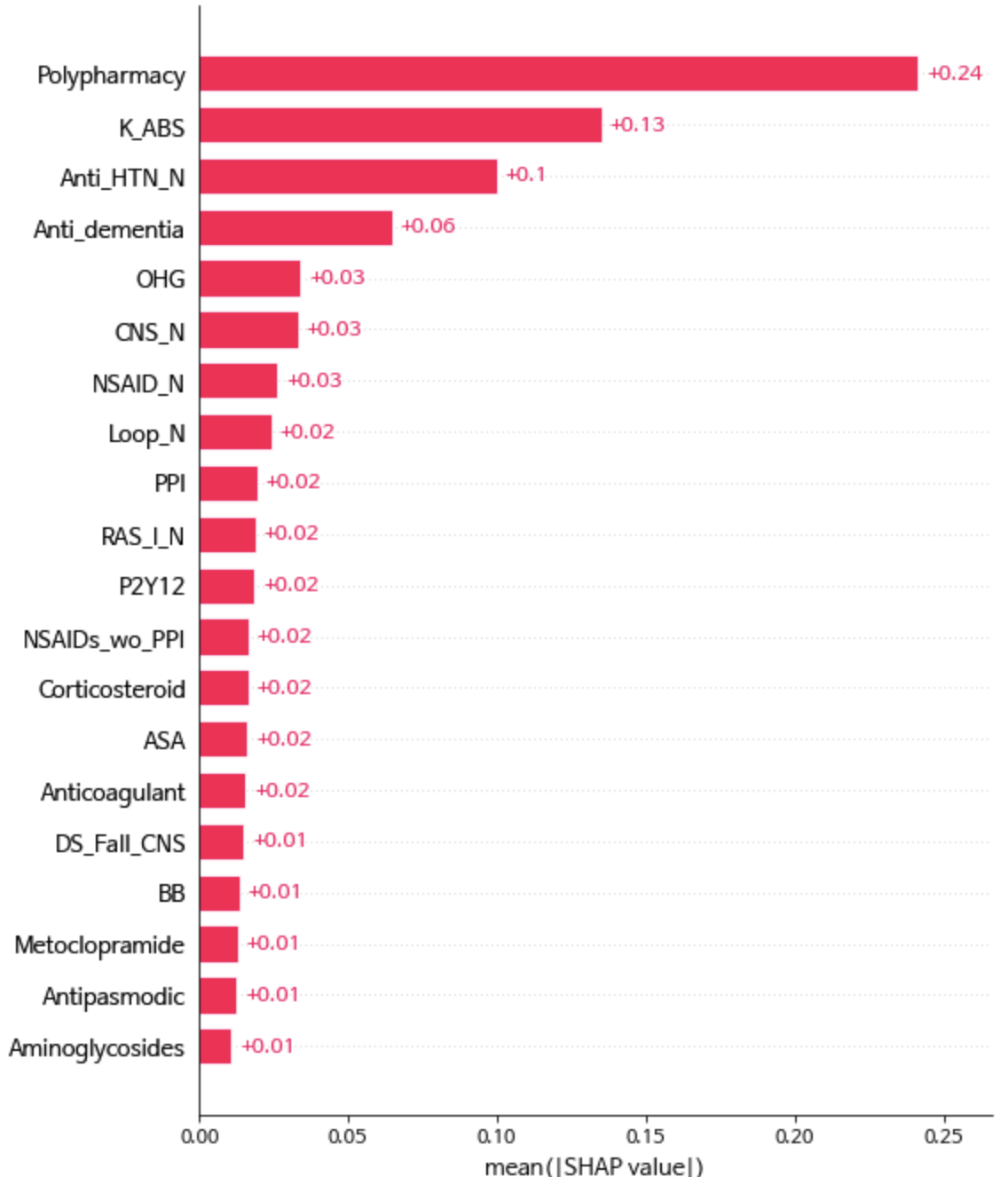

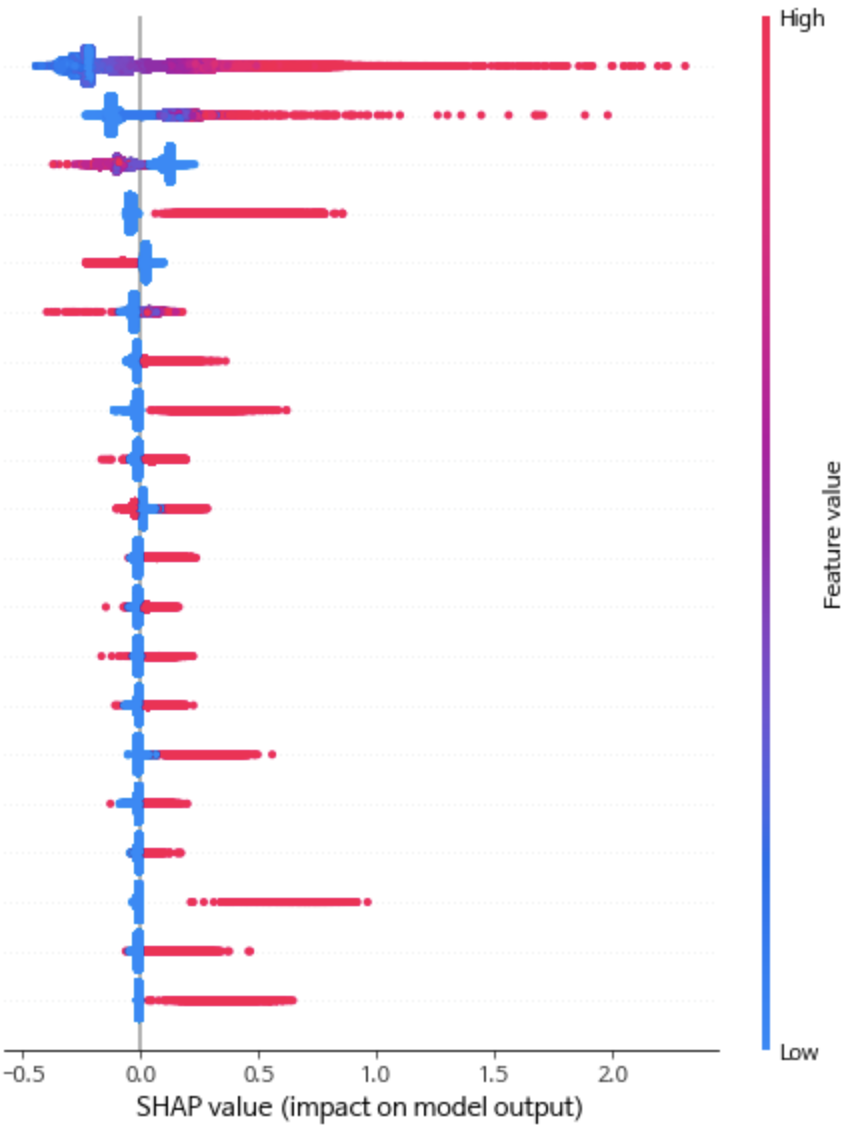


**(d)**


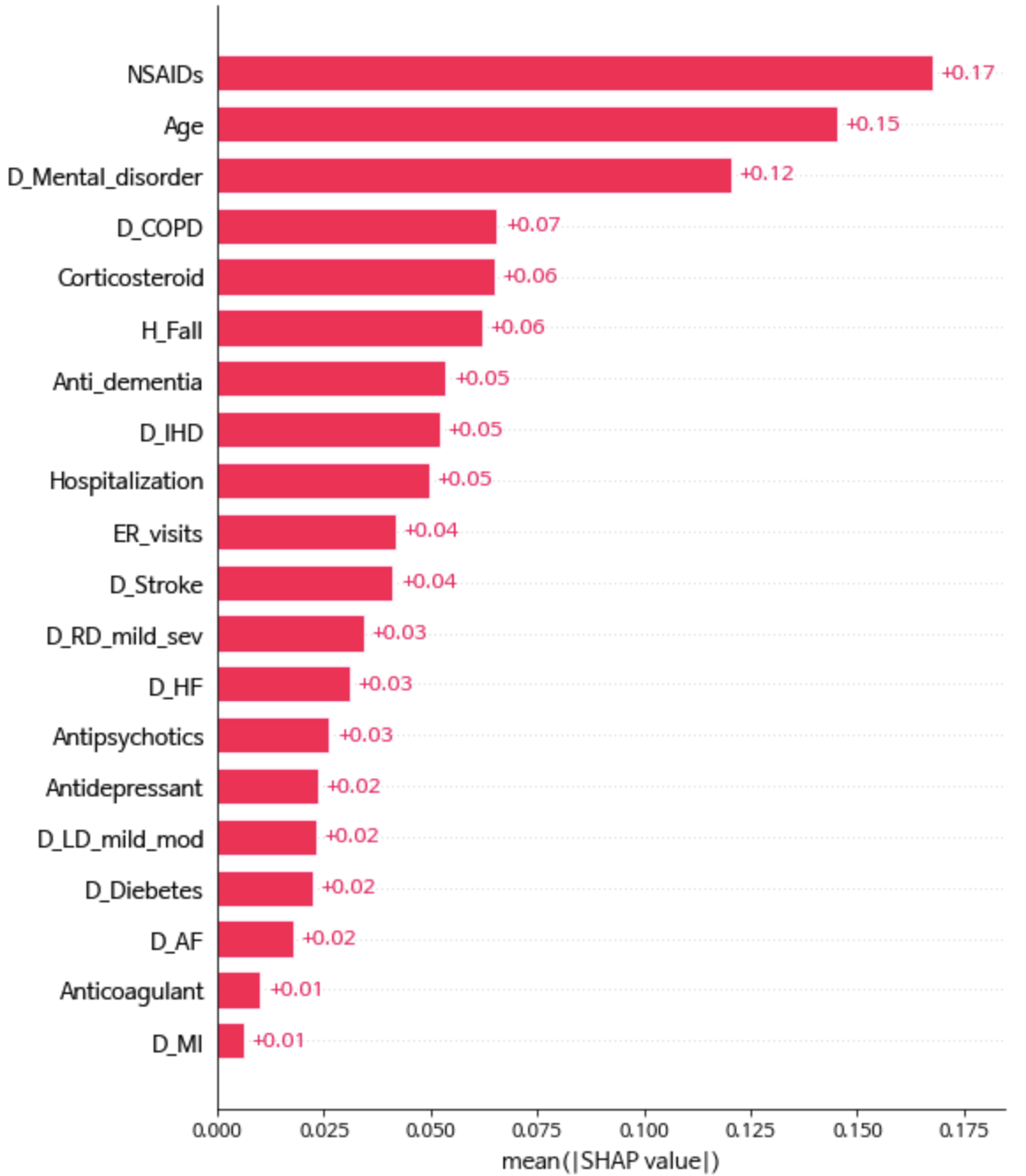

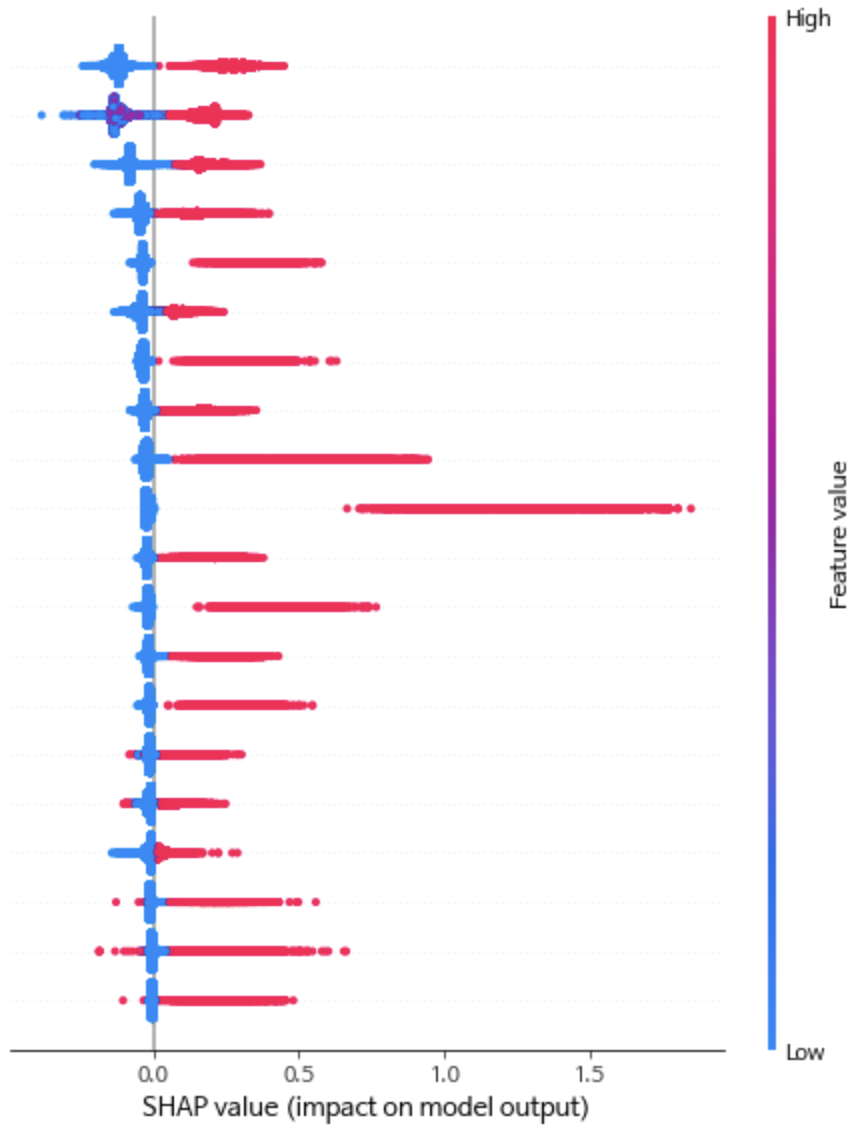


**(e)**


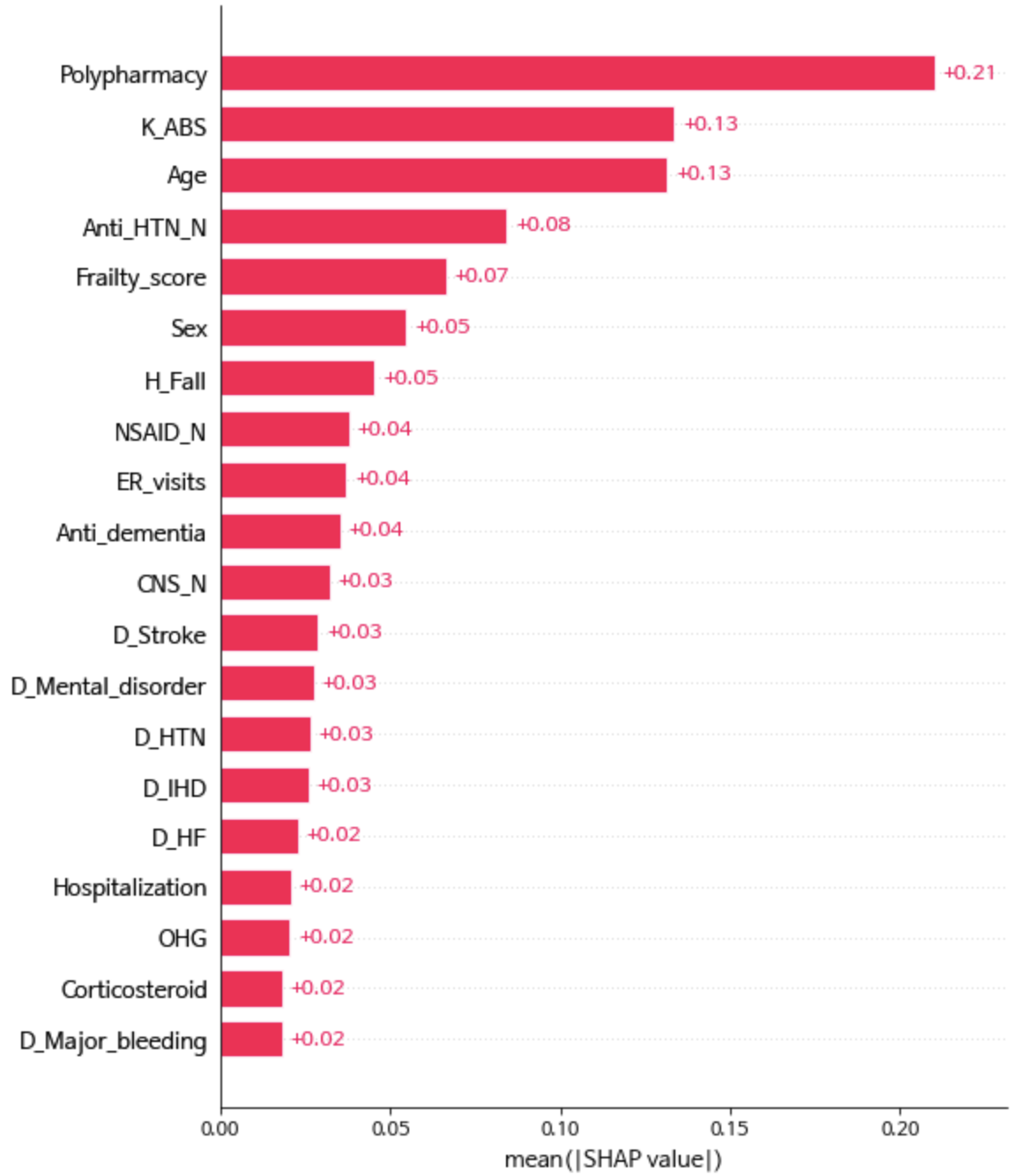

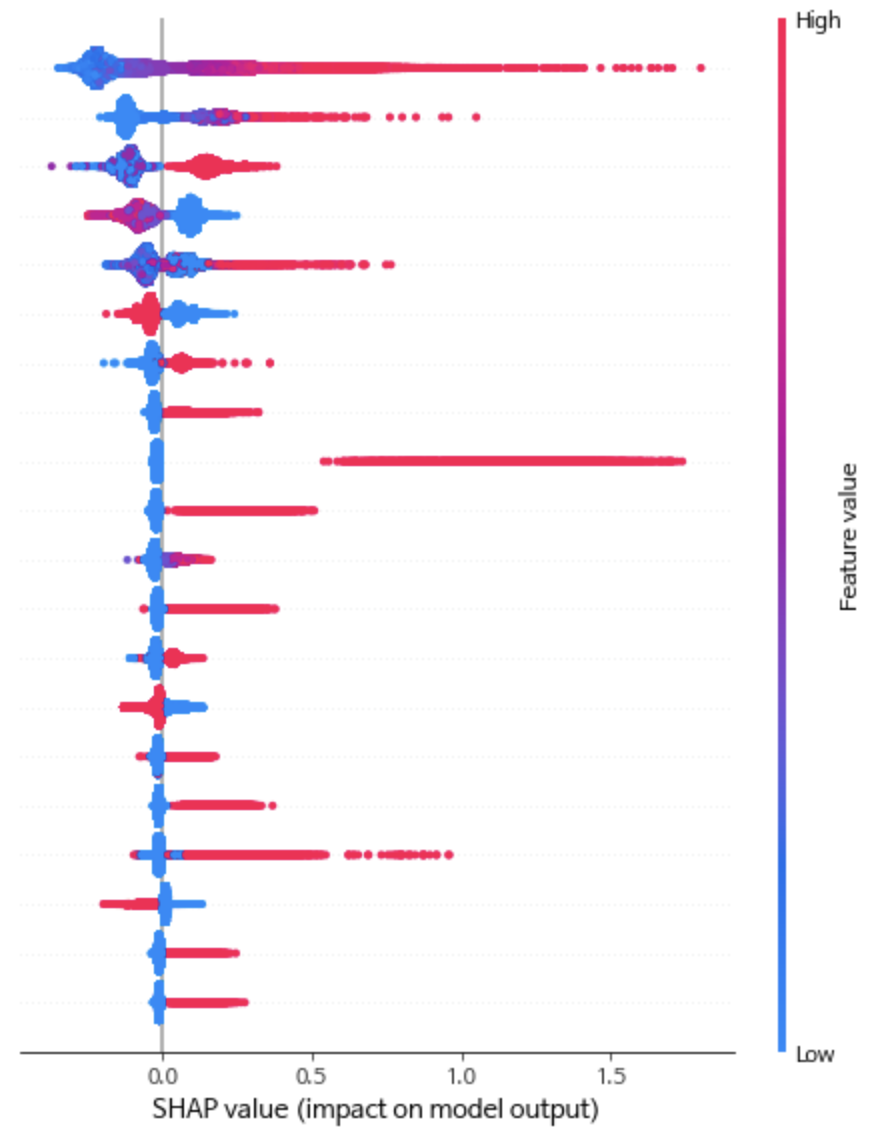


**Supplementary figure 1. Quantification of feature impact on prediction through analysis of Shapley additive explanations (SHAP) values of the 20 most impactful features (a) Sociodemographic and comorbidity; (b) Potentially inappropriate medications; (c) Medication use; (d) Baseline model; (e) Full model;**
